# The prevalence of social care in US health care settings depends on how and whom you ask

**DOI:** 10.1186/s12913-020-05338-8

**Published:** 2020-05-29

**Authors:** Yuri Cartier, Laura Gottlieb

**Affiliations:** Social Interventions Research and Evaluation Network, University of California, San Francisco, UCSF, 3333 California St, Suite 465, San Francisco, CA 94118 USA

**Keywords:** Social determinants of health, Social risk screening, Social care

## Abstract

**Background:**

Despite unprecedented enthusiasm for integrating social risk screening and related interventions into US health care settings, we know relatively little about the extent to which these activities occur. We reviewed results from multiple national surveys that reported on the prevalence of social care activities.

**Methods:**

We used snowball sampling to solicit input from 29 expert informants who were asked to share any knowledge about survey instruments that included questions on the prevalence of social care-related activities conducted in health care settings. We subsequently ran web searches on recommended surveys to identify those fielded with a national sample and conducted between Jan 1, 2007 and May 31, 2019. Finally, we analyzed and compared results across surveys.

**Results:**

We reviewed 23 total survey events (19 individual surveys and 4 that had been re-administered) that included questions on the extent of social care activities across health care disciplines and settings. Samples included a wide range of health care stakeholders (including payers, health care executives, providers, and patients.) Sample sizes ranged across the types of respondents: 95–120 respondents in surveys of payers; 44–757 in surveys of health care delivery leaders; 484–2333 in surveys of clinicians; and 500–7002 in surveys of patients. In eight cases, survey reports did not include response rates; another four reports described response rates under 25%. Fifteen of the 23 surveys incorporated questions on the prevalence of social risk screening; 17 included questions on social care intervention activities. Responses about the prevalence of both screening and interventions varied widely: between 15 and 100% of respondents reported their organization conducts screening for at least one social risk; 18–100% of respondents reported providing social care interventions. Between 3 and 22% of surveyed patients reported being screened or assisted with a social risk. In the four surveys that were administered in different years, we found no significant differences in results between survey administrations.

**Conclusions:**

Findings suggest that caution is warranted in interpreting survey findings from any single survey since existing surveys report a wide range of prevalence estimates for social risk screening and interventions.

## Background

Against the backdrop of an increasing number of value-based care initiatives [1, 2] and a strong and compelling body of evidence linking patients’ social risks such as food insecurity, housing instability, transportation barriers, or energy insecurity to health outcomes [3–6], it is not surprising that the US health care sector is turning to addressing social risk factors as one component of more comprehensive strategies to improve population health [7–10]. Interest in this area has grown sufficiently to spur a National Academies of Sciences, Engineering, and Medicine (NASEM) Committee on Integrating Social Care into the Delivery of Health Care [11]. Multiple programs at the Centers for Medicare & Medicaid Services (CMS) and state Medicaid agencies incorporate social risk factor screening and services into care innovations [12, 13]. Consequently, social risk screening tools have proliferated [14], and a new technology industry has emerged to more efficiently bridge medical providers with community and government social services [15].

Despite the unprecedented enthusiasm for integrating social risk screening and related interventions into health care delivery settings, we know relatively little about the extent to which these activities occur around the US. What we currently know about the prevalence of social care is largely derived from papers reflecting the findings of single surveys, typically focused on a particular discipline (e.g. pediatrics) [16] and/or setting (e.g. inpatient care) [17]. These surveys are used in different venues to indicate that social risk screening is either common [18] or rare [19, 20] across the health care sector. The validity of these estimates is unknown.

Improved methods for gauging the prevalence of social care activities across disciplines and settings will help target new and pending federal, state, and local policy changes that incentivize social care initiatives, such as emerging state Medicaid agency and Medicare managed care regulatory changes that have increased opportunities to cover social services [21, 22]. We undertook this review of survey results to understand the landscape of existing data on the prevalence of social care activities in health care and to explore strategies for synthesizing data across sources.

## Methods

As part of a broader research project on social risk-related activities in health care settings, between November 2017 and May 2018 we reached out to 14 experts, including from medical professional associations with published statements on social screening or interventions (the American Academy of Family Physicians and the American Academy of Pediatrics); researchers who had published articles in the academic literature on social care interventions or who had worked on national health surveys; and foundations funding projects on health care sector social care initiatives. Thirteen of the initial 14 agreed to participate in a 30–60 min semi-structured interview. As part of the interview, we asked participants to share information about survey initiatives that had been conducted with health care-affiliated stakeholders (including providers and leaders in organizations that deliver health care services, administer health plans/insurance, and/or provide other services related to medical care) about the prevalence of social risk screening or interventions, or that had asked patients if they had received screening or assistance in their health care setting. We defined health-care based social risk screening as a health care activity related to assessing patients’ social circumstances (e.g. food security, housing stability, transportation access). We defined social care interventions as health care-initiated programs or services intended to mitigate or address social risks, either directly (e.g. by offering food or transportation) or indirectly (e.g. by providing assistance with connecting to food benefits programs or supportive housing services). The initial 13 experts were asked to recommend additional informants with expertise in this area. Using this snowball sampling strategy, we spoke with 29 experts, stopping interviews when no new survey initiatives were mentioned by additional informants.

**Fig. 1:**
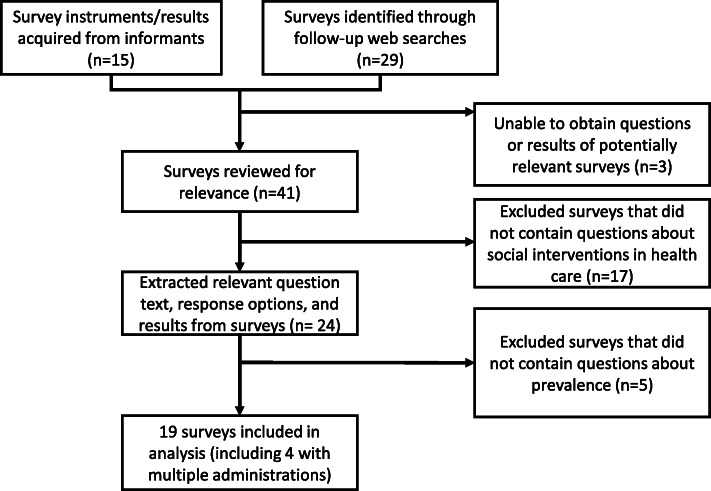
Diagram of survey identification and inclusion process

We subsequently ran Google web searches on the 15 surveys mentioned during the 29 interviews to identify surveys that were conducted Jan 1, 2017 or later, were fielded with a national sample, and included questions related to the prevalence of social care activities in the US health care sector. The web searches were used to locate the fielded survey instruments and/or published survey results. We sent email requests to survey-fielding organizations when we were unable to find either full text surveys or a description of survey results online. Both the Health Resources & Services Administration (HRSA) Bureau of Primary Health Care and the Commonwealth Fund provided full data sets, which enabled us to conduct additional analyses published elsewhere [23, 24]. We also used references included in published survey results to find additional reports and publications about surveys that met our inclusion criteria. These follow-up searches yielded an additional 29 surveys (Fig. 1). Surveys that were limited to local, regional, or state respondents were excluded. Though our intent was to exclude surveys that had not been conducted with a national sample, included surveys were not required to be nationally representative.

From each included survey, we extracted basic information on survey characteristics, including survey-fielding organization, survey title, year administered, sampling frame, response rate and number of respondents. Additionally, we extracted question text and results for questions pertaining to social care activities. Survey items fell into two clusters: 1. Questions related to screening patients for social risks; and 2. Questions related to providing social services or related interventions. We subsequently excluded surveys that did not include questions about the prevalence of social risk screening or social care-related interventions. Taking into consideration the most frequently-queried social risk domains in included surveys, we compared questions and results in surveys with similar target populations (e.g. patients, clinical providers, etc.) across the five domains included in the CMS Accountable Health Communities health-related social needs screening tool as well as non-domain specific questions. Differences in survey respondents and questions/response options prohibited quantitative pooling of the reported prevalence of these activities. Instead, we conducted a qualitative review of findings across surveys conducted with similar target populations.

## Results

We collected information on 19 surveys that had been conducted across multiple states with groups of health care stakeholders (including state Medicaid agencies, payers, health care executives, providers, and patients) and that incorporated questions about the prevalence of health care-based social risk screening and related social care programs [25]. Table 1 describes the characteristics of the 19 included surveys. Four surveys were administered twice, for a total sample of 23 survey events. Sample sizes ranged across the types of respondents: 95–120 respondents in surveys of payers; 44–757 in surveys of health care delivery leaders, 484–2333 in surveys of clinicians, and 500–7002 in surveys of patients.

**Table 1:** Surveys on prevalence of social risk screening and/or social care interventions^a^

Category	Survey-fielding organization (year of administration)	Sample characteristics	Sample size (response rate)
Payers	Change Healthcare & HealthCare Executive Group (2018) [26]	Private payers and healthcare executives (N= > 2000)	*n* = 120 (6%). Of the respondents, 54% represented health plans.
	Change Healthcare & HealthCare Executive Group (2019) [27]	Private payers and healthcare executives (N= > 2000)	Not reported.
	Institute for Medicaid Innovation (2019) [28]	Medicaid Managed Care Organizations	n not reported, though report describes that sample represents 69% of Medicaid managed care covered lives
	Kaiser Family Foundation (2017) [29]	Medicaid Managed Care Organizations (*N* = 277)	*n* = 95 plans (34%), representing 31 of 39 states.
Health Care Delivery Systems	America’s Essential Hospitals Essential Hospitals Institute (2016) [30]	Safety-net hospitals (*N* = 108 systems, representing 242 hospitals)	*n* = 44 systems (41%) representing 109 hospitals (42%)
	American Pediatrics Association Continuity Research Network (2017) [31]	Pediatric resident continuity clinics (*N* = 158)	*n* = 65 (41%)
	Children’s Hospital Association (2015) [32]	Children’s hospitals (*N* = 207)	*n* = 73 (35%)
	Commonwealth Fund (2013) [33]	FQHCs (*N* = 1128)	*n* = 679 (60%)
	Commonwealth Fund (2018) [34, 35]	FQHCs (*N* = 1367)	*n* = 694 (51%)
	Dartmouth Institute for Health Policy & Clinical Practice (2018) ^b^ [17]	Hospitals (*N* = 1628)	*n* = 757 (47%)
	Deloitte Center for Health Solutions (2017) [36]	Hospitals and health systems (*N* = 4257)	*n* = 284 (22%)
	National Center for Medical-Legal Partnerships (2016) [37, 38]	Health care organizations participating in MLPs (*N* = 266)	*n* = 128 (48%)
	Numerof & Associates (2018) [39]	Health care organization executives (*N* = 9600)	*n* = 411 (4.3%)
Providers	American Academy of Pediatrics (2014) [16]	Pediatricians (N = approx. 1500)	*n* = 708 (47%)
	American Association of Family Physicians (2017) [40]	Family physicians (*N* = 5000)	*n* = 484 (10%)
	Commonwealth Fund (2012) [41]	Primary care physicians in 11 countries, including the US (*N* = 3067)	2012: US: *n* = 1012 (33%)
	Commonwealth Fund (2015) [24, 42]	Primary care physicians in 11 countries, including the US (*N* = 2567)	2015: US: *n* = 1001 (39%)
	Dartmouth Institute for Health Policy & Clinical Practice (2018) [17]	Physician practices (*N* = 4976).	*n* = 2333 (47%)
	Leavitt Partners Physician Survey (2018) [43]	Active physicians (N not provided)	*n* = 550; response rate unknown
Patients/Consumers	Health Resources and Services Administration (HRSA) Bureau of Primary Health Care (2009) [23]	Health center patients (N not provided)	*n* = 4562 patients from 112 health centers
	HRSA Bureau of Primary Health Care (2014) [23]	Health center patients (N not provided)	*n* = 7002 patients from 169 health centers
	Leavitt Partners Consumer Survey (2018) [43]	Adults 18 or over in the US (N not provided)	*n* = 5006; response rate unknown
	National Council on Aging (2014) [44]	Older adults (N not provided)	*n* = 3279; response rate unknown
	Waystar (2018) [45]	Consumers (N not provided)	n = 500; response rate unknown

^a^Four surveys were administered twice—one time/year in two different years. Each administration is listed separately since response rates (and in some cases questions) differed across administrations

^b^The Dartmouth Institute for Health Policy & Clinical Practice (2018) survey is listed twice as results were reported separately for hospital/system respondents and physician practice respondents

*FQHC* Federally Qualified Health Center

Seven excluded surveys incorporated questions related to staffing, data collection, or attitudes about social care but did not include questions about the prevalence of screening or interventions. [Information about those surveys is available in Supplemental Table 1]. Fifteen of the 23 surveys included questions about screening activities; 17 included questions about social interventions (nine included questions on both topics).

Information about the frequency of domains included in the survey questions is presented in Supplemental Tables 2 and 3. Compared to surveys including items about social care interventions, surveys that only included items about social risk screening included more detail (i.e., regarding specific social risk domains). Food insecurity was the most commonly included social domain in surveys that included questions about social risk screening (*n* = 8, 53%); transportation was the most commonly included domain surveys that included questions about social care interventions (n = 8, 47%). The 5 domains used in the CMS Accountable Health Communities Social Needs screening tool were among the most frequently referenced domains overall; employment and education also appeared in approximately one-third of surveys.

### Prevalence of social risk screening

Table 2 presents results from surveys that included a question about the prevalence of social risk screening, e.g. “Do you screen your patients for social needs?” Across the 15 survey administrations that included screening prevalence questions, question structure and content both varied. Nine surveys asked about social risk screening generally [26, 27, 29, 32, 37, 38, 40, 45]; eight asked respondents to indicate specific social risk domains included in screening activities [16, 17, 28, 30, 31, 34, 36, 43]. One survey presented screening as an answer option to a question about population health activities [30].

**Table 2:** Range of respondents reporting social risk screening activities in each survey category

	Any social risk	Food	Housing	Transportation	Utilities	IPV
Payer surveys (total n = 4)	15% [26]	100% [28]	100% [28]	86% [28]	79% [28]	86% [28]
	15% [27]					
	91% [29]					
Health care delivery system surveys (total n = 7)	62% [36]	32% [30]	50% [34]	27% [30]	29% [34]	46% [30]
	69% [32]	40% [17]	57% [30]	47% [34]	36% [17]	49% [31]
	79% [38]	42% [34]	60% [17]	68% [36]	40% [36]	57% [34]
	91% [17]	67% [36]	70% [36]	74% [17]		75% [36]
		71% [31]				76% [17]
Provider surveys (total n = 3)	59% [40]	30% [17]	28% [17]	35% [17]	23% [17]	57% [17]
	67% [17]	52% [16]	53% [16]	68% [16]	44% [16]	
Patient/consumer surveys (total n = 2)	22% [45]	6% [43]	8% [43]	6% [43]	4% [43]	

The five social risk domains most commonly included under screening activities were food security, housing stability, transportation access, utility needs, and interpersonal violence. Rates of survey respondents endorsing screening activities in each of these categories across the surveys are presented in Table 2. Only two surveys also reported the overall prevalence of screening for at least one social risk domain. Three surveys that included social risk screening questions included response options about the frequency of screening. In papers about survey results, answer options were typically dichotomized (e.g., *any* screening vs. *no* screening; or *frequent and sometimes* vs. *occasional and no* screening).

Depending on the survey, between 15% [26] and 100% [28] of respondents reported their organization conducts screening for at least one social risk. There were substantial differences in reported prevalence of screening by social risk domain (e.g. food security screening ranged from 30% [17] to 100% [28]; transportation access screening ranged from 27% [30] to 86%) [28].

When clustered by category of respondent (e.g. patient, provider, clinic, hospital) results still ranged widely across surveys. For example, in the four hospital or system-level survey administrations reporting on screening for any social risk, the results ranged from 62 [36] to 91% [17]. Results for food security screening in this group ranged from 32% [30] to 71% [31]; for transportation, from 27% [30] to 74% [17]; and for IPV, results ranged from 46% [30] to 76% [17]. Variability between surveys was lower for screening for housing issues (50% [34] to 70% [36]), and for utility security (ability to pay for utilities) (29% [34] to 40% [36]).

Twenty-one of the 23 surveys provided no information about the percent of the total population served who were screened for social risk. Exceptions included one consumer survey that asked patients if their health care provider had discussed social needs with them; 22% of patients reported that this had occurred in the last 12 months [45]. A second exception was a survey of community health center leaders, which asked about the proportion of the total served patient population that was screened for social needs. In this survey, 40% of respondents reported screening all patients and 49% reported screening only some patients [34].

### Prevalence of social care interventions

Table 3 displays the prevalence of intervention activities in the 17 surveys that included questions about the prevalence of social care interventions. Similar to the variation in social risk screening questions, survey questions about interventions differed significantly in content and structure. Some surveys asked about the specific type of intervention provided, such as availability of a database of community social service resources or referrals to social service providers; others asked more generally about any activity that addressed a specific social risk (e.g. interventions around food security). As with the questions about screening, answer options across surveys differed, as did ways responses were dichotomized in reported results.

**Table 3:** Respondents reporting social intervention activities (including referrals) in each survey category

	Any social risk	Food	Housing	Transportation	Utilities	IPV
Payer surveys (n = 4)	18% [27]	73% [29]	77% [29]	79% [28]	100% [28]	79% [28]
	19% [26]	100% [28]	100% [28]			
	93% [29]					
Health care delivery system surveys (n = 5)	52% [36]	64% [30]	68% [30]	31% [33]	43% [30]	75% [30]
	54% [34]			45% [34]		
	70% [39]			61% [30]		
Provider surveys (*n* = 5)	52% [40]	27% [43]	20% [43]	35% [43]	22% [43]	45% [43]
	91% [41]	66% [16]	23% [16]	50% [16]	25% [16]	
	92% [42]					
Patient/consumer surveys (n = 3)	19% [46]	5% [23]	3% [23]	10% [23]		
				11% [23]		

Depending on the survey, 18 [27]–100% [28] of respondents indicated that their organization provides social care interventions. The two extreme percentages were reported by the same two payer surveys that reported lowest and highest prevalence of screening. Among surveys conducted in health care delivery systems, overall social care intervention prevalence varied from 52% [36] to 70% [39]; in clinical provider surveys, the range was 52% [40] to 92% [42]. As in the case of social risk screening, surveyed patients reported significantly lower rates of interventions than providers or health system leaders (19% among a sample of older adults [46] and between 5 and 11% of health center patients reporting assistance, depending on the social need [23].)

In the four surveys that included the same questions in repeated administrations (Table 4), there were no statistically significant changes in reported prevalence across administrations. The 15 other surveys either had not been repeated or had not included comparable questions in repeated administrations.

**Table 4:** Response trends for survey questions with multiple administrations

Survey name	Question (Definition of positive response)	First administration	Second administration	Difference	***p***^***a***^
Industry Pulse	How is your organization integrating social determinants of health into your population health programs? --Coordinating with community programs and resources (Yes)	19.30% (2017) [26]	18.40% (2018) [27]	−0.90%	0.82^b^
Commonwealth Fund FQHC	How often do patients at your largest site receive the following services when they need them: --Transportation (Usually or Often)	48.36% (2013) [33]	45% (2018) [35]	−3.36%	0.21
Commonwealth Fund IHPS	Do you and/or other personnel that work with you in your practice provide care in any of the following ways? – Coordinate care with social services or other community providers (2012: Yes; 2015: Frequently or Occasionally)	92.30% (2012) [24, 41]	91.00% (2015) [24, 42]	−1.30%	0.29
HRSA Patient Survey	Has anyone at {the reference health center} ever helped you with transport to medical appointments? (Yes)	10.10% (2009) [23]	10.50% (2014) [23]	0.40%	0.49

^a^ Calculated with two-sided z test

^b^ We were unable to obtain a sample size for the 2018 Industry Pulse survey; the *p*-value is therefore calculated from a conservative estimate of 300% of the 2017 sample size

*FQHC* Federally qualified health center; *HRSA* Health Resources and Services Administration; *IHPS* International Health Policy Survey

## Discussion

There is no question that the language of social determinants is in vogue in the US health care sector. But how much, where, and when the health care system currently invests in identifying, helping to address, and tracking patients’ social risks is unknown. This is the first study of which we are aware that looks across different survey initiatives to estimate the prevalence of social care activities conducted in health care settings. In our review of 19 different surveys and 23 survey events, we found wide variation in reported rates of social risk screening and related interventions.

Differences in the reported frequencies of social risk screening and intervention activities across surveys could stem from true practice variation. For example, Medicaid managed care organization samples reported nearly universal rates of social risk screening and intervention, whereas surveys of mostly private payer samples reported prevalence of social risk activities below 20%. In delivery settings, it is possible that social risk screening is conducted more frequently during hospitalizations than in outpatient community health center settings. It also is conceivable that safety-net hospitals, though they serve more disadvantaged populations, are less likely to have the resources to conduct social risk assessments than better-resourced private hospital facilities. These findings, however, suggest that social care is less likely to be provided to patients more likely to need it.

An alternative explanation is that differences in reported frequencies derive from the challenges of designing surveys to adequately capture this information. Only three surveys indicate the reach of social risk screening or interventions by providing an indication of the denominator for patients offered social care services (i.e. total served patients who are screened or who receive services). In other words, survey respondents might endorse having social care programs even if those programs are limited to one setting or even one group of patients, e.g. patients with diabetes attending group visits, rather than available to the entire served population. At the system level, denominators also differ across surveys. For example, the one survey targeting safety-net hospital leaders used skip logic in asking about social risk screening; those who had responded affirmatively to a question about select population health activities were asked the social risk screening question [30]. The lack of a consistent denominator constrains comparisons across surveys.

All of the surveys rely on self-report, in many cases by a single executive responding for an entire organization that may have multiple clinical sites with substantial variations in practice. As a result, it is possible that the reports from patients about their own experiences are a more accurate representation of practice.

Our results highlight common challenges across survey initiatives, underscoring several barriers to using surveys to gauge social care across different settings. Stakeholders interested in any one discipline (e.g. family medicine), population (e.g. older adults), or setting (e.g. community health centers) could—and do—extract findings from the most relevant surveys and critically interpret those findings in light of the limitations of that particular dataset [16, 17]. Given the gaps in available information and differences across surveys conducted even in similar populations, however, these data are unlikely to be useful for targeting efforts to scale effective programs.

Developing a more accurate and generalizable estimate of the extent to which social care activities—including both screening and interventions—are incorporated into clinical care in the US at minimum will require more standardization in the surveys being used to establish prevalence. This could be facilitated by developing and testing a core set of meaningful and precise survey questions. If survey-sponsoring organizations could achieve consensus on a validated common core of questions, it would improve the reliability and comparability of survey findings. Developing a common core would require deepening a national dialogue about the goals of social screening and social interventions. For example, currently there seems to be no agreement on whether questions about screening should inquire about social screening of any kind occurring in a given setting or screening for specific risks, such as housing or food. Moving forward, initiatives that use surveys to examine trends in screening and intervention adoption over time should include denominator information in survey questions, e.g. by asking about the proportion of the patient population being screened. Given that social care activities related to food, housing, transportation, utilities, and IPV are included in federal demonstration projects and common to many common screening tools, future national surveys on prevalence should consider including these core domains at minimum.

Surveys may not be the most accurate gauge of clinical activities. Another option would be for federal and state agencies to develop and encourage use of documentation standards for social care, including social risk screening, assessment and diagnosis, and treatment/counseling/referrals, in electronic health records. Standardized documentation could strengthen efforts to track these activities and enable data aggregation across systems. To make coding feasible would require overcoming a new set of challenges, such as the availability of accurate codes and incentives around documentation [47, 48]. It is possible that in the near future natural language processing algorithms applied to electronic health records (EHRs) will automate these coding processes by finding relevant information in EHR notes and using that information to standardize documentation [49].

### Limitations

We relied on national key informant experts using snowball sampling to identify survey instruments that attempted to estimate the prevalence of social care in the US health care sector. We selected this method after consulting with an academic librarian and conducting multiple pilot searches of academic databases that did not adequately surface relevant publications. Our search strategy enabled us to include surveys where findings were not intended for academic or other publication. It is nonetheless possible that informants were unfamiliar with relevant surveys. Additionally, in some cases, neither the survey questions nor results were yet available to include in this synthesis. There is no reason to assume the common limitations we found in the study sample (e.g. low sample size, low response rates, respondent bias) would be overcome in other surveys, though if considering a common question core across future survey initiatives, it would be appropriate to review survey questions from all available sources. Lastly, we did not include regional or state-specific surveys, nor did publications on included surveys conduct region-specific analyses. This precluded us from identifying potential geographic variability in social risk screening or social care intervention practices.

## Conclusions

There is wide variation in the results of surveys intended to gauge the prevalence of social care provided in US health care settings. As the health care sector debates new investments in this area, more careful attention should focus on how to estimate program depth and breadth. Only then can we understand the effectiveness of policy and practice incentives that aim to scale social care programs.

## Supplementary information

**Additional file 1: Supplemental Table 1**. Surveys excluded from analysis. **Supplemental Table 2.** Number of surveys that include questions about screening for social (and behavioral) risks. **Supplemental Table 3.** Number of surveys that include questions about interventions related to social (and behavioral) risks.

## Data Availability

The datasets used and/or analyzed during the current study are available from the corresponding author on reasonable request.

## Abbreviations

CMS: Centers for Medicare & Medicaid Services
EHR: Electronic health record
FQHC: Federally qualified health center
HRSA: Health Resources & Services Administration
IHPS: International Health Policy Survey
MLP: Medical-legal partnership
NASEM: National Academies of Sciences, Engineering, and Medicine
US: United States
