# Supplementary material for: The prevalence of social care in US health care settings depends on how and whom you ask
Source: BMC Health Serv Res. 2020 May 29;20:481. doi: 10.1186/s12913-020-05338-8 (PMC7260787; doi:10.1186/s12913-020-05338-8)
Supplement: Supplementary file 1 — Additional file 1: Supplemental Table 1. Surveys excluded from analysis. Supplemental Table 2. Number of surveys that include questions about screening for social (and behavioral) risks. Supplemental Table 3. Number of surveys that include questions about interventions related to social (and behavioral) risks. [file 12913_2020_5338_MOESM1_ESM.docx]

Supplemental tables

**Supplemental Table 1. Surveys excluded from analysis**

| **Survey name** | **Sponsor** |
| --- | --- |
| HFMA Value Based Readiness Survey (2017) | HFMA and Humana |
| Survey of Medicaid managed care organizations (2018) | Institute for Medicaid Innovation |
| Medicaid Budget Survey for State Fiscal Years 2017 and 2018 (2017) | Kaiser Family Foundation |
| Physician Survey on Social Determinants of Health (2017) | Leavitt Partners |
| Leavitt Partners Consumer Survey (2017) | Leavitt Partners |
| Leavitt Employer Survey (2017) | Leavitt Partners |
| Survey of Medicaid Medical Directors (2017) | Ohio State University |
| Health Care’s Blind Side (2011) | Robert Wood Johnson Foundation |

**Supplemental Table 2. Number of surveys that include questions about screening for social (and behavioral) risks**

| **Domain** | **N (Total N = 15)** | **%** |
| --- | --- | --- |
| Food insecurity | 8 | 53% |
| Housing instability or quality | 7 | 47% |
| Transportation (medical or nonmedical) | 7 | 47% |
| Utility needs | 7 | 47% |
| Interpersonal violence | 6 | 40% |
| No domains (e.g. Do you screen for social needs? Y/N) | 6 | 40% |
| Education | 5 | 33% |
| Employment/skills training | 5 | 33% |
| Mental illness (incl. depression) | 3 | 20% |
| Family/social support/social isolation | 3 | 20% |
| Low health literacy | 2 | 13% |
| Substance use disorder | 2 | 13% |
| Financial support/strain | 2 | 13% |
| Parental stress | 2 | 13% |
| Medicaid eligibility | 1 | 7% |
| Need for financial assistance with medical bills | 1 | 7% |
| Opioid use | 1 | 7% |
| Polypharmacy | 1 | 7% |
| Tobacco use | 1 | 7% |
| Child maltreatment | 1 | 7% |
| Childcare needs | 1 | 7% |
| Community infrastructure | 1 | 7% |
| Environmental health | 1 | 7% |
| Financial literacy | 1 | 7% |
| Firearm exposure | 1 | 7% |
| Home environment | 1 | 7% |
| Immigration | 1 | 7% |
| Incarceration | 1 | 7% |
| Neighborhood environment | 1 | 7% |
| Trauma | 1 | 7% |

**Supplemental Table 3. Number of surveys that include questions about interventions related to social (and behavioral) risks**

| **Domain** | **n (N = 17)** | **%** |
| --- | --- | --- |
| No domains (e.g. Do you refer patients to CBOs for social needs? Y/N) | 8 | 47% |
| Transport | 8 | 47% |
| Food | 7 | 41% |
| Housing | 7 | 41% |
| Employment/skills training/income | 7 | 41% |
| Utilities | 5 | 29% |
| Education | 5 | 29% |
| IPV | 4 | 24% |
| Family/social support/social isolation | 4 | 24% |
| Public benefits | 4 | 24% |
| Childcare needs | 2 | 12% |
| Financial literacy | 1 | 6% |
| Environmental health | 1 | 6% |
| Trauma | 1 | 6% |
| Community infrastructure | 1 | 6% |
| Patient/parental stress | 1 | 6% |
| Mental illness (incl. depression) | 1 | 6% |
| Medicaid eligibility/insurance | 1 | 6% |
| Early childhood development | 1 | 6% |
| Free medication | 1 | 6% |
| Basic needs | 1 | 6% |
| Clothes/shoes | 1 | 6% |
